# Diagnostic performance of PET, SPECT and CMR perfusion imaging for the detection of significant coronary artery disease - a meta-analysis

**DOI:** 10.1186/1532-429X-13-S1-P75

**Published:** 2011-02-02

**Authors:** Caroline Jaarsma, Tim Leiner, Sebastiaan Bekkers, Harry Crijns, Joachim Wildberger, Eike Nagel, Patricia Nelemans, Simon Schalla

**Affiliations:** 1 Maastricht University Medical Center, Maastricht, Netherlands; 2 University Medical Center Utrecht, Maastricht, Netherlands; 3 King's College, London, UK

## Context

Non-invasive myocardial perfusion imaging for the detection of significant coronary artery disease (CAD) has advanced rapidly. Consensus on the best imaging modality has not been reached as few studies compare them directly.

## Objective

To determine and compare the diagnostic accuracy of positron emission tomography (PET), single-photon emission computed tomography (SPECT) and cardiac magnetic resonance (CMR) perfusion imaging in the diagnosis of CAD.

## Data sources

Studies published in the English language between January 1980/1990 (for PET / SPECT and MRI respectively) and February 2010 identified by PubMed search and citation tracking were examined.

## Study selection

A study was included if PET, SPECT or CMR perfusion imaging was used as a diagnostic test for the detection of CAD (≥50% diameter stenosis) and used coronary angiography as the reference standard.

## Data extraction

Four reviewers screened potential studies and independently extracted study data. Discrepancies were resolved by consensus.

## Data synthesis

Of the 3694 citations, 166 articles (17.744 patients) met the inclusion criteria: 18 PET (1.441 patients), 111 SPECT (13.462 patients) and 37 CMR (2.841 patients) articles respectively. The overall patient based analysis demonstrated a sensitivity of 0.85 (95% confidence interval [CI] 0.83-0.88), 0.88 (95% 0.88-0.89) and 0.89 (95% CI 0.88-0.91); and a specificity of 0.83 (95% CI 0.76-0.88), 0.60 (95% CI 0.59-0.62), 0.76 (95% CI 0.73-0.78) for PET, SPECT and MRI respectively.

## Conclusion

Non-invasive PET, SPECT or CMR perfusion imaging are accurate modalities for the detection of CAD. Of the three different imaging modalities PET has the best diagnostic performance. However, the number of publications on PET perfusion imaging is limited and the prevalence of CAD in PET study populations was higher. In addition, this technique is hampered in daily practice by availability and costs. CMR provides an excellent alternative without ionizing radiation exposure and a similar diagnostic accuracy.

